# Biological Anti-TNF-*α* Therapy and Markers of Oxidative and Carbonyl Stress in Patients with Rheumatoid Arthritis

**DOI:** 10.1155/2021/5575479

**Published:** 2021-12-22

**Authors:** Emőke Šteňová, Martina Bakošová, Lucia Lauková, Peter Celec, Barbora Vlková

**Affiliations:** ^1^1st Department of Internal Medicine, Faculty of Medicine, Comenius University and University Hospital, Bratislava, Slovakia; ^2^Institute of Molecular Biomedicine, Faculty of Medicine, Comenius University, Bratislava, Slovakia; ^3^Department for Biomedical Research, Center for Biomedical Technology, Danube University Krems, Krems, Austria; ^4^Institute of Pathophysiology, Faculty of Medicine, Comenius University, Bratislava, Slovakia; ^5^Department of Molecular Biology, Faculty of Natural Sciences, Comenius University, Bratislava, Slovakia

## Abstract

Rheumatoid arthritis (RA) as a chronic inflammatory disease is associated with oxidative stress. Drugs targeting tumor necrosis factor-alpha (TNF-*α*) ameliorate inflammation and symptoms of RA in most patients. Whether markers of oxidative stress can be used for monitoring of treatment effects is unknown. The aim of our study was to analyze the effects of anti-TNF-*α* treatment on oxidative stress in plasma and saliva of patients with RA. Samples were collected from 26 patients with RA at baseline as well as 3 and 6 months after starting the anti-TNF-*α* treatment. Thiobarbituric acid-reacting substances (TBARS), advanced oxidation protein products (AOPP), advanced glycation end products (AGEs), and fructosamine were quantified using spectrophotometry and spectrofluorometry in plasma. TBARS were measured also in saliva. The disease activity score (DAS28) was used to assess the clinical status of patients. No significant dynamic changes were found except plasma TBARS that decreased continuously. At 6 months after starting the treatment, plasma TBARS were lower by 39% in comparison to baseline (*p* = 0.006). Salivary concentrations of TBARS did not reflect the dynamics in plasma. Although a trend was observed (*r* = 0.33), a significant correlation between plasma TBARS and DAS28 was not found. Our results indicate that anti-TNF-*α* treatment decreases plasma TBARS as a marker of lipid peroxidation. However, the lack of a significant correlation with DAS28 suggests that it cannot be used for monitoring of treatment. Other markers of oxidative stress and antioxidant capacity with lower biological variability should be tested in future studies.

## 1. Introduction

Rheumatoid arthritis (RA) is a chronic inflammatory disease [1]. The inflammation is not restricted to the joints; it is rather systemic [2]. Patients with RA have a reduced life expectancy mainly due to atherosclerosis and its complications [3]. Atherogenic LDL cholesterol particles are chemically modified to be recognized by macrophages [4]. The modifications are often mediated by oxidative or carbonyl stress [5].

Oxidative stress is a dysbalance between the production of reactive oxidants and antioxidant mechanisms [6]. The overproduction of free radicals or a lower production of antioxidants leads to oxidation of various macromolecules, especially lipids and proteins. Chronic inflammation is a common cause of oxidative stress [7, 8]. Activated immune cells, mainly neutrophils and macrophages, enzymatically produce reactive oxygen species to fight against bacteria and other microorganisms [9]. However, in case of chronic sterile inflammation, the prolonged overproduction of reactive oxygen species leads to oxidative damage of tissues [10].

Carbonyl stress is best known for its role in the pathogenesis of diabetic complications [11]. The nonenzymatic glycation is dependent on the concentrations of glucose or other glycating agents [12]. However, oxidative stress-induced lipid peroxidation leads to the production of malondialdehyde and other carbonyls that stimulate carbonyl stress. The end products are a heterogenous group of compounds called advanced glycation end products (AGEs) which act proinflammatory closing the vicious circle [13].

RA is associated with oxidative stress as shown in previously summarized studies [14]. Among the oxidative stress markers, thiobarbituric acid-reacting substances (TBARS) are most widely used to assess lipid peroxidation in RA patients [15]. Advanced oxidation protein products (AOPP) as a marker of protein oxidation were also found to be higher in RA patients [16]. Similarly, AGEs as a nonspecific marker of carbonyl stress and fructosamine as a precursor of AGEs are higher in RA than in controls [17, 18].

Biological drugs targeting tumor necrosis factor-alpha (TNF-*α*) ameliorate inflammation and symptoms of RA with a much higher efficiency and safety than any other treatment in the past [19]. However, whether the anti-inflammatory treatment also reduces oxidative or carbonyl stress is not clear. Similarly, it is not clear which marker of oxidative or carbonyl stress is most sensitive to the reduction of inflammation. This could be of importance for the understanding of the pathogenesis of RA and also for the potential interventions to prevent its complications.

The aim of our study was to analyze the effects of anti-TNF-*α* treatment on selected markers of oxidative and carbonyl stress in plasma and saliva of patients with RA. We hypothesized that the treatment would decrease lipid peroxidation early and other markers later during the treatment. In addition, we expected that salivary TBARS could be a noninvasive alternative that reflects plasma TBARS dynamics as a marker of RA activity.

## 2. Material and Methods

### 2.1. Patients

The study included 26 adult patients with RA who were naïve with regard to biological anti-TNF-*α* treatment. The patients received monoclonal antibodies or decoy receptors targeting TNF-*α*: adalimumab (*n* = 18, 40 mg s.c. every other week), golimumab (*n* = 3, 50 mg s.c. once a month), certolizumab (*n* = 3, 200 mg s.c. every other week), or etanercept (*n* = 2, 50 mg s.c. once a week). Exclusion criteria included major comorbidities such as cancer or heart failure, as well as treatment with anti-TNF-*α* drugs in the past. Disease activity score of 28 joints (DAS28) was used to assess the severity of the disease in RA patients. The basic characteristics of the cohort of patients are summarized in Table 1. All patients were followed during the whole duration of the study.

### 2.2. Blood and Saliva Collection

Blood samples were collected into EDTA tubes, and unstimulated saliva samples were collected into sterile tubes in 3 time points: before as well as 3 and 6 months after starting the biological anti-TNF-*α* treatment. Samples were centrifuged at 1600*g* for 5 min, and supernatants were stored at -20°C until analyzed.

### 2.3. Measurement of Markers of Oxidative and Carbonyl Stress

Thiobarbituric acid-reacting substances (TBARS) as a marker of lipid peroxidation were measured as previously described [20]. Plasma and saliva samples were mixed with thiobarbituric and acetic acids, which formed colored complexes after incubation at 94°C for 45 minutes. The samples were then cooled to 4°C. Afterwards, n-butanol was added and the mixture was shaken continuously for 2 minutes. Phase separation was performed by centrifugation at 2000*g* for 10 minutes. Upper phase was afterwards carefully removed and measured at 553 nm emission and 515 nm excitation wavelength. TBARS contents were quantified based on calibration curve made using 1,1,3,3-tetramethoxypropane.

The marker of protein oxidation—advanced oxidation protein products (AOPP)—was determined using spectrophotometry after addition of phosphate-buffered saline to the samples and exposure to glacial acetic acid for 2 minutes. The mixture of chloramine T and potassium iodine was used for construction of calibration curve. Specific absorbance was taken at 340 nm [21].

For measurement of advanced glycation end products (AGEs), as markers of protein glycation, samples were diluted in phosphate-buffered saline (1 : 10) and calibrated with AGE-modified bovine serum albumin. The absorbance of prepared reaction mixture was immediately measured at 440 nm emission and 370 nm excitation wavelength, respectively [22].

Fructosamine was assessed by measuring the absorbance at 530 nm after addition of nitro blue tetrazolium solution (mixture of nitro blue tetrazolium and sodium carbonate buffer, pH = 10.35) into the samples and incubation for 15 minutes at 37°C [23]. To prepare calibration curve, the mixture of 1-deoxy-morpholino-D-fructose, sodium chloride, and albumin was used.

The concentration of proteins in plasma and saliva was estimated by commercially available bicinchoninic acid assay (BCA, Fermentas, Lithuania). The assessed concentrations of oxidative and carbonyl stress markers were normalized to proteins. All measurements were performed using spectrofluorometer Sapphire II (Tecan, Austria). All chemicals and reagents used in these experiments were purchased from Sigma-Aldrich (Germany).

### 2.4. Statistical Analysis

All data were analyzed using GraphPad Prism 6.0 (La Jolla, California, USA) software using Friedman test for nonparametric datasets with Dunn's multiple comparison test. Correlations between DAS28 and TBARS were assessed using the Pearson correlation test. Differences were considered significant when *p* < 0.05.

## 3. Results

**Figure 1:**
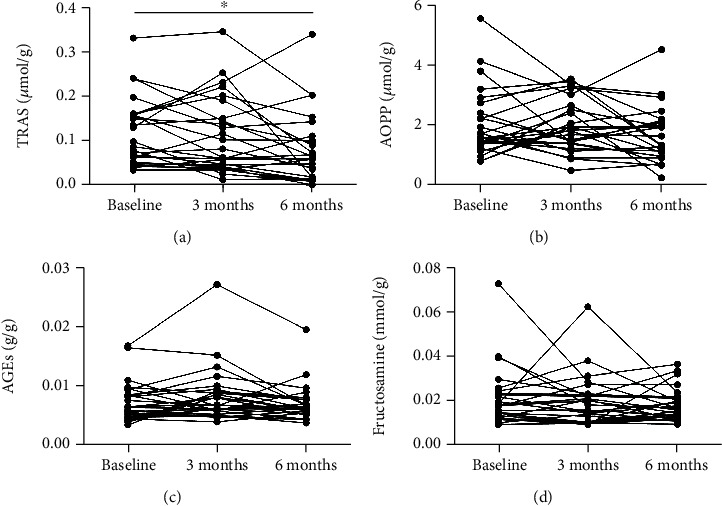
Concentration of (a) thiobarbituric acid-reacting substances (TBARS), (b) advanced oxidation protein products (AOPP), (c) advanced glycation end products (AGEs), and (d) fructosamine in plasma of patients with rheumatoid arthritis before and after anti-TNF-*α* treatment. Differences between time points were assessed using repeated measures one-way ANOVA test and Bonferroni's multiple comparisons test (*n* = 26). ^∗^*p* < 0.05.

**Figure 2:**
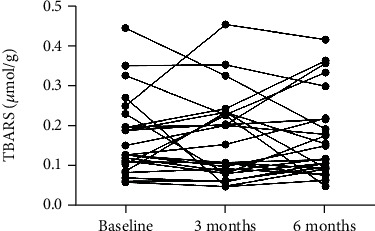
Concentration of thiobarbituric acid-reacting substances (TBARS) in saliva of patients with rheumatoid arthritis before and after anti-TNF-*α* treatment. Differences between time points were assessed using repeated measures one-way ANOVA test and Bonferroni's multiple comparison test (*n* = 26).

**Figure 3:**
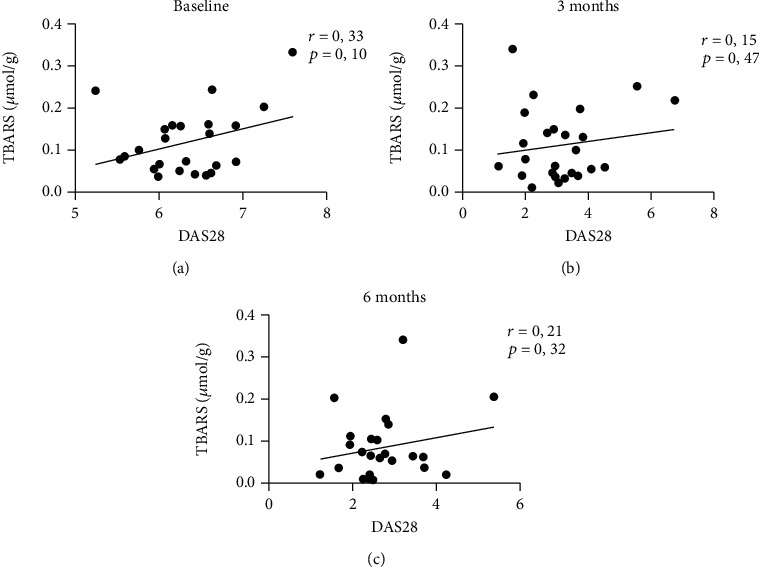
Correlations between disease activity score of 28 joints (DAS28) and thiobarbituric acid-reacting substances (TBARS) in plasma of patients with rheumatoid arthritis before and after anti-TNF-*α* treatment. Correlations were assessed using the Pearson correlation test (*n* = 26).

The results showed that the median concentration of TBARS in plasma of patients with RA decreased by 12% 3 months after starting anti-TNF-*α* therapy (from 0.098 to 0.080 *μ*mol/g, *F* = 8.9, *p* = 0.41). Significantly lower concentration (by 39%) of TBARS was assessed 6 months after the beginning of treatment (to 0.060 *μ*mol/g, Figure 1(a), *p* = 0.006). AOPP in plasma of patients with RA decreased by 17% 6 months after start of anti-TNF-*α* therapy without reaching statistical significance (from 1.60 to 1.33 *μ*mol/g, Figure 1(b), *F* = 2.2, *p* = 0.41). Concentration of AGEs in plasma samples stayed without major changes 3 as well as 6 months after beginning of anti-TNF-*α* treatment in comparison with baseline median values (Figure 1(c), *F* = 5.4, *p* = 0.96 for 3 months and *p* = 0.24 for 6 months). A slight decrease of median fructosamine concentrations by 6% at 6 months after biological treatment was observed in plasma of patients with RA. This difference was not significant (from 0.017 to 0.016 mmol/g, Figure 1(d), *F* = 1.7, *p* = 0.41). No differences were observed in salivary concentration of TBARS 3 as well as 6 months after starting anti-TNF-*α* treatment (0.128, 0.131, and 0.134 *μ*mol/g; Figure 2, *F* = 0.6, *p* = 0.99). Correlation analysis revealed positive but not significant correlations between DAS28 and concentration of TBARS in plasma of RA patients before the start of treatment (Figure 3(a), *p* = ns, *r* = 0.33). The correlations between DAS28 and TBARS decreased 3 and 6 months after administration of anti-TNF-*α* treatment (Figures 3(b) and 3(c), *p* = *ns*, *r* = 0.47 and 0.32).

## 4. Discussion

The results of our study show that the lipid peroxidation marker TBARS is decreased by the anti-inflammatory treatment, not the protein oxidation marker AOPP or fructosamine and AGEs as markers of carbonyl stress. This could be explained by the fact that lipids are more sensitive to oxidative damage than proteins [24]. It is likely that after a longer observation protein oxidation and carbonylation might decrease as well. Indeed, a study focusing on a specific AGE molecule—pentosidine—showed that it decreased after nearly one year of anti-TNF-*α* treatment [25]. Our results are in line with the published data on reactive oxygen species production, which decreased after 6 months and after 12 months of treatment with anti-TNF-*α* [26]. In our study, we have not measured the reactive oxygen species, but the damage caused by oxidative stress. This includes the protective effect of total antioxidant status scavenging the free radicals.

Oxidative stress is present in RA patients, and it is associated with the risk of atherosclerosis [27]. The fact that anti-inflammatory treatment decreases a biomarker of oxidative damage is compatible with the hypothesis that oxidative stress is a consequence of the chronic inflammation in RA. This does not support the use of antioxidants as adjuvants to the anti-inflammatory treatment suggested previously [28]. Our study does not support the use of TBARS as a marker of treatment effects in RA patients as it decreased very slowly. The lack of a clear significant correlation between TBARS and DAS28 is also in line with published data. Patients with RA were shown to have higher TBARS than controls, but patients with a higher DAS28 score and, thus, a worse clinical status did not differ significantly from patients with lower DAS28 scores [29].

This study is the first to analyze the effects of anti-TNF-*α* on both plasma and salivary TBARS. The results show that while plasma TBARS decreased, salivary TBARS did not. The origin of salivary TBARS is unclear—it might be diffusion from plasma, but salivary TBARS could also be produced directly in the oral cavity. Our previous research indicated that salivary TBARS might be of local oral rather than systemic origin [30]. On the other hand, we have also shown that some systemic pathologies might be reflected by higher salivary TBARS as shown for multiple sclerosis [31]. These contradictory findings might have a simple explanation—the association between salivary and plasma TBARS is context dependent and varies between patient groups.

RA is associated with periodontitis [32]. While the causality of this association and its direction are unclear, it is suggested that chronic untreated or treatment-resistant periodontitis can induce RA in genetically predisposed individuals [33]. Periodontal pathogens stimulate immune cells, especially neutrophils, to produce extracellular traps as a major driver of the pathogenesis of the chronic systemic inflammation and local joint symptomatology in RA [34]. Salivary TBARS increased due to periodontal inflammation could, thus, reflect the local oral health status. However, this was not monitored in our study. On the other hand, it has been shown that periodontal status in RA patients is improved by anti-TNF-*α* treatment as a wanted side effect [35, 36]. The lack of effects of the treatment on salivary TBARS in our study requires further analyses.

Our study suffers from several limitations. These include the use of nonspecific biomarkers of oxidative stress, which are, however, very sensitive and should change quickly as a consequence of oxidative imbalance. The cohort is rather small, and a larger study with more statistical power would be needed if subtle effects would be of interest. In addition, longer follow-up of the patients combined with oral health monitoring would be needed to better understand the dynamics of salivary biomarkers of oxidative stress.

One potential approach to study the effects of the anti-inflammatory treatment on biomarkers could be the use of animal models. Collagen-induced arthritis in DBA/1J mice has been used to study the effects of a CXCR3 antagonist on numerous markers of the cellular and humoral immune response [37, 38]. A similar experiment focusing on the selected markers of oxidative and carbonyl stress combined with the analysis of expression of relevant genes in immune cell populations could bring a novel mechanistic insight with potential translation to human patients [39, 40]. In addition, the use of prepared collagen antibodies enables the use of other mouse strains and, thus, also genetic models [41].

In conclusion, this study shows that anti-TNF-*α* treatment decreases lipid peroxidation without affecting protein oxidation or carbonyl stress. Plasma TBARS cannot be used for monitoring of treatment efficiency because they do not reflect the clinical status. Future studies and experiments should focus on other more specific markers with lower biological variability. Whether the induced changes in plasma TBARS have any relevance for the pathogenesis of atherosclerosis in RA remains to be elucidated.

## Figures and Tables

**Table 1:** Patient characteristics. Median (interquartile range) is reported for the quantitative variables. BMI: body mass index; CRP: C-reactive protein; ESR: erythrocyte sedimentation rate.

	All	Men	Women
Number (*n*)	26	9	17
Age (years)	58.5 (46-65.5)	59 (44-69)	58 (55-64)
BMI (kg/m^2^)	24.9 (22.2-26.6)	25.6 (24.2-26.6)	24.1 (22.0-26.2)
Methotrexate (*n*)	19/26	7/9	12/17
Corticoids (*n*)	14/26	5/9	9/17

All patients	Baseline	After 3 months	After 6 months
DAS28 [1]	6.4 (6.1-6.7)	3.0 (2.1-3.6)	2.7 (2.3-3.4)
CRP < 5 mg/l (*n*)	1/26	15/26	16/26
CRP (mg/l)	18.1 (11.6-30.9)	13.0 (7.7-30.3)	7.7 (6.1-22.9)
ESR (mm/1^st^ hour)	48 (30-59)	—	20 (13.3-33.5)

## Data Availability

The data used to support the findings of this study are available from the corresponding author upon request.

## References

[1] Smolen J. S., Aletaha D., Barton A. (2018). Rheumatoid arthritis. *Nature Reviews. Disease Primers*.

[2] Figus F. A., Piga M., Azzolin I., McConnell R., Iagnocco A. (2021). Rheumatoid arthritis: extra-articular manifestations and comorbidities. *Autoimmunity Reviews*.

[3] Skeoch S., Bruce I. N. (2015). Atherosclerosis in rheumatoid arthritis: is it all about inflammation?. *Nature Reviews Rheumatology*.

[4] Summerhill V. I., Grechko A. V., Yet S. F., Sobenin I. A., Orekhov A. N. (2019). The atherogenic role of circulating modified lipids in atherosclerosis. *International Journal of Molecular Sciences*.

[5] Stocker R., Keaney J. F. (2004). Role of oxidative modifications in atherosclerosis. *Physiological Reviews*.

[6] Pizzino G., Irrera N., Cucinotta M. (2017). Oxidative stress: harms and benefits for human health. *Oxidative Medicine and Cellular Longevity*.

[7] Lugrin J., Rosenblatt-Velin N., Parapanov R., Liaudet L. (2014). The role of oxidative stress during inflammatory processes. *Biological Chemistry*.

[8] Marchio P., Guerra-Ojeda S., Vila J. M., Aldasoro M., Victor V. M., Mauricio M. D. (2019). Targeting early atherosclerosis: a focus on oxidative stress and inflammation. *Oxidative Medicine and Cellular Longevity*.

[9] Ndrepepa G. (2019). Myeloperoxidase - a bridge linking inflammation and oxidative stress with cardiovascular disease. *Clinica Chimica Acta*.

[10] de Almeida A., de Almeida Rezende M. S., Dantas S. H. (2020). Unveiling the role of inflammation and oxidative stress on age-related cardiovascular diseases. *Oxidative Medicine and Cellular Longevity*.

[11] Miyata T., Ishikawa N., Strihou C. V. (2003). Carbonyl stress and diabetic complications. *Clinical Chemistry and Laboratory Medicine*.

[12] Ahmed N., Thornalley P. J. (2007). Advanced glycation endproducts: what is their relevance to diabetic complications?. *Diabetes, Obesity & Metabolism*.

[13] Ott C., Jacobs K., Haucke E., Navarrete Santos A., Grune T., Simm A. (2014). Role of advanced glycation end products in cellular signaling. *Redox Biology*.

[14] Quiñonez-Flores C. M., González-Chávez S. A., Del Río Nájera D., Pacheco-Tena C. (2016). Oxidative stress relevance in the pathogenesis of the rheumatoid arthritis: a systematic review. *BioMed Research International*.

[15] Mititelu R. R., Pădureanu R., Băcănoiu M. (2020). Inflammatory and oxidative stress markers-mirror tools in rheumatoid arthritis. *Biomedicine*.

[16] Baskol G., Demir H., Baskol M. (2006). Investigation of protein oxidation and lipid peroxidation in patients with rheumatoid arthritis. *Cell Biochemistry and Function*.

[17] Babu N. P., Bobby Z., Selvaraj N., Harish B. N. (2006). Increased fructosamine in non-diabetic rheumatoid arthritis patients: role of lipid peroxides and glutathione. *Clinical Chemistry and Laboratory Medicine*.

[18] de Groot L., Hinkema H., Westra J. (2011). Advanced glycation endproducts are increased in rheumatoid arthritis patients with controlled disease. *Arthritis Research & Therapy*.

[19] Conti F., Ceccarelli F., Massaro L. (2013). Biological therapies in rheumatic diseases. *La Clinica Terapeutica*.

[20] Behuliak M., Pálffy R., Gardlík R., Hodosy J., Halcák L., Celec P. (2009). Variability of thiobarbituric acid reacting substances in saliva. *Disease Markers*.

[21] Witko-Sarsat V., Friedlander M., Capeillère-Blandin C. (1996). Advanced oxidation protein products as a novel marker of oxidative stress in uremia. *Kidney International*.

[22] Münch G., Keis R., Wessels A. (1997). Determination of advanced glycation end products in serum by fluorescence spectroscopy and competitive ELISA. *European Journal of Clinical Chemistry and Clinical Biochemistry*.

[23] Chung H. F., Lees H., Gutman S. I. (1988). Effect of nitroblue tetrazolium concentration on the fructosamine assay for quantifying glycated protein. *Clinical Chemistry*.

[24] Fritz K. S., Petersen D. R. (2011). Exploring the biology of lipid peroxidation-derived protein carbonylation. *Chemical Research in Toxicology*.

[25] Kageyama Y., Takahashi M., Ichikawa T., Torikai E., Nagano A. (2008). Reduction of oxidative stress marker levels by anti-TNF-alpha antibody, infliximab, in patients with rheumatoid arthritis. *Clinical and Experimental Rheumatology*.

[26] Cacciapaglia F., Anelli M. G., Rizzo D. (2015). Influence of TNF-*α* inhibition on oxidative stress of rheumatoid arthritis patients. *Reumatismo*.

[27] da Fonseca L. J. S., Nunes-Souza V., Goulart M. O. F., Rabelo L. A. (2019). Oxidative stress in rheumatoid arthritis: what the future might hold regarding novel biomarkers and add-on therapies. *Oxidative Medicine and Cellular Longevity*.

[28] van Vugt R. M., Rijken P. J., Rietveld A. G., van Vugt A. C., Dijkmans B. A. (2008). Antioxidant intervention in rheumatoid arthritis: results of an open pilot study. *Clinical Rheumatology*.

[29] Veselinovic M., Barudzic N., Vuletic M. (2014). Oxidative stress in rheumatoid arthritis patients: relationship to diseases activity. *Molecular and Cellular Biochemistry*.

[30] Celec P., Hodosy J., Celecová V. (2005). Salivary Thiobarbituric Acid Reacting Substances and Malondialdehyde – Their Relationship to Reported Smoking and to Parodontal Status Described by the Papillary bleeding index. *Disease Markers*.

[31] Karlík M., Valkovič P., Hančinová V., Krížová L., Tóthová Ľ., Celec P. (2015). Markers of oxidative stress in plasma and saliva in patients with multiple sclerosis. *Clinical Biochemistry*.

[32] Bartold P. M., Lopez-Oliva I. (2020). Periodontitis and rheumatoid arthritis: an update 2012-2017. *Periodontology 2000*.

[33] Potempa J., Mydel P., Koziel J. (2017). The case for periodontitis in the pathogenesis of rheumatoid arthritis. *Nature Reviews Rheumatology*.

[34] de Molon R. S., Rossa C., Thurlings R. M., Cirelli J. A., Koenders M. I. (2019). Linkage of periodontitis and rheumatoid arthritis: current evidence and potential biological interactions. *International Journal of Molecular Sciences*.

[35] Cotti E., Mezzena S., Schirru E. (2018). Healing of apical periodontitis in patients with inflammatory bowel diseases and under anti-tumor necrosis factor alpha therapy. *Journal of Endodontia*.

[36] Fabri G. M., Pereira R. M., Savioli C. (2015). Periodontitis response to anti-TNF therapy in ankylosing spondylitis. *Journal of Clinical Rheumatology*.

[37] Bakheet S. A., Alrwashied B. S., Ansari M. A. (2020). CXCR3 antagonist AMG487 inhibits glucocorticoid-induced tumor necrosis factor- receptor-related protein and inflammatory mediators in CD45 expressing cells in collagen-induced arthritis mouse model. *International Immunopharmacology*.

[38] Bakheet S. A., Ansari M. A., Nadeem A. (2019). CXCR3 antagonist AMG487 suppresses rheumatoid arthritis pathogenesis and progression by shifting the Th17/Treg cell balance. *Cellular Signalling*.

[39] Ansari M. A., Nadeem A., Bakheet S. A. (2021). Chemokine receptor 5 antagonism causes reduction in joint inflammation in a collagen-induced arthritis mouse model. *Molecules*.

[40] Bakheet S. A., Alrwashied B. S., Ansari M. A. (2020). CXC chemokine receptor 3 antagonist AMG487 shows potent anti-arthritic effects on collagen-induced arthritis by modifying B cell inflammatory profile. *Immunology Letters*.

[41] Abd-Allah A. R., Ahmad S. F., Alrashidi I. (2014). Involvement of histamine 4 receptor in the pathogenesis and progression of rheumatoid arthritis. *International Immunology*.

